# LSTM–Transformer-Based Mine Pressure Prediction Using Hydraulic-Support Monitoring Data

**DOI:** 10.3390/s26144423

**Published:** 2026-07-12

**Authors:** Ran Tao, Xiaowan Lei, Lirong Wan, Yan Wang, Nan Xu

**Affiliations:** College of Mechanical and Electronic Engineering, Shandong University of Science and Technology, Qingdao 266590, China; taoran@sdust.edu.cn (R.T.); wanlr666@163.com (L.W.); yanwang@sdust.edu.cn (Y.W.); ranehale@sdust.edu.cn (N.X.)

**Keywords:** mine pressure prediction, LSTM–transformer, longwall mining, time-series forecasting, intelligent mining

## Abstract

Accurate mine pressure prediction is essential for understanding roof–support interaction and supporting intelligent monitoring in fully mechanized longwall mining. In underground production, hydraulic-support pressure sensors provide continuous pressure sequences that reflect the mechanical response of the support–roof system. However, these sequences are affected by local mining disturbances, missing records, abnormal zero-value segments, nonstationary fluctuations, and periodic weighting, which make future pressure forecasting challenging. To address this issue, an LSTM–Transformer hybrid model is proposed for hydraulic-support pressure forecasting. The LSTM module extracts local nonlinear pressure-evolution features from recent historical windows, whereas the Transformer module captures temporal dependencies and periodic pressure patterns through global sequence modeling. Support-wise experiments were conducted using field monitoring data from Yili No. 1 Mine, and the pressure sequence of each support was processed independently to avoid mixing information from different support locations. In the representative test case, the proposed model achieved an R^2^ of 0.971 and reduced the MAE to 0.471 MPa, while improving the phase consistency of predicted pressure peaks. Further analysis indicates that sufficient historical data coverage is necessary to capture complete pressure-evolution cycles, and that the 25-step forecasting case maintains stable accuracy for short-term mine pressure estimation. These findings demonstrate the feasibility of the proposed approach for hydraulic-support pressure prediction under the monitored conditions of the studied working face.

## 1. Introduction

As coal mining extends to deeper seams, the spatial structure of overlying strata in the stope becomes increasingly complex, and intense mine pressure has become a major constraint on safe and efficient coal production. As the core load-bearing equipment in the human–machine–environment interaction system of a fully mechanized longwall face, hydraulic supports provide a direct and measurable reflection of roof movement through variations in working resistance and column pressure. Therefore, accurate prediction of future mine pressure evolution is important for understanding roof–support interaction, evaluating support status, and supporting intelligent roof-control decisions.

With the development of intelligent coal mines, massive support-pressure monitoring data provide new opportunities to overcome the limitations of traditional semi-empirical strata-control theories. Compared with manual observations or isolated measurements, continuous pressure sensor records can describe the dynamic response of hydraulic supports at higher temporal resolution. Data-driven mine pressure prediction based on machine learning and deep learning has therefore become an important research direction at the intersection of mining engineering, sensing technology, and artificial intelligence.

Extensive studies have been conducted worldwide in the fields of strata pressure manifestation, slope instability, dynamic disasters, and related engineering safety prediction. Yin Xiwen et al. [1] proposed a dual-cycle analysis and prediction method for strata pressure, establishing a high-precision model from both temporal and spatial dimensions to achieve real-time prediction. In slope and landslide prediction, Li Yun et al. [2] combined ICEEMDAN decomposition with Attention-LSTM, effectively improving the prediction accuracy of slope deformation. For coal mine dynamic disasters, Pu Yuanyuan et al. [3] developed a full spatiotemporal control optimization system based on digital twins. Zhao Yixin et al. [4] employed an LSTM-based deep learning model to predict strata pressure patterns on fully mechanized faces with large mining height and further enhanced model generalization through transfer learning, thereby constructing and validating a strata pressure prediction system. Gu Yufei et al. [5] integrated an improved LSTM with PID-MPC to achieve high-accuracy air-conditioning prediction and energy-saving control. Cheng Jingyi et al. [6] developed an intelligent strata pressure perception system based on online monitoring data from hydraulic supports on a fully mechanized longwall face, enabling roof weighting prediction, roof-fall risk identification, and support-state evaluation, and establishing an intelligent support–roof perception framework for the stope. Xu Gang et al. [7] presented a calculation method for strata pressure evolution and a support-crushing criterion for top-coal caving faces, clarifying the safety risks caused by insufficient support working resistance. Wang et al. [8] combined grey theory with a BP neural network and proposed an improved grey neural network model, which effectively enhanced the prediction accuracy of mine roof pressure while reducing training time. Xu Huicong et al. [9] addressed the insufficient accuracy of strata pressure prediction for shallow-buried western working faces by combining the “inclined step rock beam” theory with an improved Transformer architecture and proposed an efficient intelligent prediction method. Lu Jie et al. [10] combined LSTM with the Nadam optimization algorithm, and the resulting Nadam–LSTM model showed higher accuracy and stability in predicting key strata pressure parameters. Cui Feng et al. [11] applied the PatchTST model to long-sequence strata pressure prediction on extended working faces and verified its good applicability. Deng Yanhui et al. [12] established a CNN-BiLSTM strata pressure prediction model capable of accurately capturing periodic characteristics of strata pressure. Zhu Sitao et al. [13] classified roof-movement-induced mine seismicity and established an energy prediction model together with a prevention and control framework. Zeng Qingtian et al. [14] proposed a Prophet+LSTM hybrid model incorporating the influence of adjacent supports, and further reduced prediction errors through data denoising and weighted fusion. Lei et al. [15] established a mechanical–hydraulic interaction model to investigate the operational reliability of hydraulic supports with joint clearances, revealing that clearance-induced dynamic effects can influence cylinder pressure fluctuations, motion accuracy, and posture stability of hydraulic supports. Ji Wenli et al. [16,17] proposed GA-GRU-BP and MBCT-SR-RF prediction models, respectively, both of which outperformed traditional BP and SVM methods in predicting strata pressure locations and roof weighting events on working faces.Yang et al. [18] introduced a time-series LSTM-based displacement prediction model, Li [19] proposed a variable time-series shift Transformer-LSTM ensemble method for mine-pressure prediction, and Gong et al. [18,19,20] developed an MRDA-FLPEM integrated algorithm for transfer prediction of mine pressure in fully mechanized working faces. Chai Jing et al. [21,22] proposed an improved GRU-GoogleNet model and a Bayesian-optimized CatBoost model, respectively, and combined them with distributed optical fiber monitoring to achieve accurate prediction of strata pressure manifestation intensity and roof weighting state. Shu Yunlong et al. [23] proposed the SCSSA-CNN-BiLSTM model, significantly improving the prediction accuracy of roof weighting interval. Liu Gan and Lou Xuan et al. [24,25] proposed LSTM-CNN and grey-LSTM dust concentration prediction models, respectively, and effectively improved dust prediction accuracy in open-pit mines through multi-algorithm fusion.

Although existing deep learning–based hybrid models have achieved promising results in mine pressure prediction and related engineering time-series tasks, several challenges remain in modeling hydraulic-support pressure sequences under complex longwall mining conditions. First, many studies still treat pressure monitoring data mainly as mathematical time-series signals, while the engineering meanings of pressure accumulation, pressure release, and pressure transfer in the roof-support system are not sufficiently discussed. Second, hydraulic-support pressure sequences contain both short-term abrupt fluctuations caused by local mining disturbances and longer-term periodic weighting processes associated with roof movement. Conventional sequence models often struggle to characterize local pressure mutations and global pressure-evolution trends simultaneously.

To address these issues, an LSTM–Transformer hybrid model is proposed for future mine pressure prediction based on hydraulic-support pressure monitoring data. The LSTM module extracts local nonlinear features from support-pressure sequences within historical input windows, and the Transformer module models temporal dependencies and periodic pressure behavior. Field-measured data from the 1503 fully mechanized working face of Yili No. 1 Mine were used to validate the feasibility and effectiveness of the proposed method. In the experiments, pressure sequences from different supports were processed independently so that the prediction task reflected support-specific temporal evolution rather than mixed spatial samples. The objective of this study is to predict future mine pressure evolution from measured pressure sensor sequences, rather than to construct an alarm-classification system. Compared with previously reported LSTM-, Transformer-, CNN-BiLSTM-, and Prophet+LSTM-based forecasting methods, the methodological novelty of this study lies in three aspects. First, the LSTM module is not used only as a general sequence-fitting tool; its gating behavior is linked to the engineering processes of pressure accumulation, pressure release, and pressure transfer in the roof-support system, thereby improving the physical interpretability of feature extraction. Second, a Transformer encoder with learnable positional embedding is introduced after LSTM-based local feature extraction, enabling the model to jointly represent short-term pressure mutations and longer-range periodic weighting behavior. Third, the pressure sequence of each hydraulic support is modeled independently rather than concatenating data from different support positions, so that the prediction task reflects support-specific temporal evolution and reduces artificial information mixing. These features distinguish the proposed method from existing hybrid time-series models and make it suitable for nonstationary hydraulic-support pressure prediction under the monitored working-face conditions.

## 2. Strata Pressure Time-Series Prediction Model Based on LSTM–Transformer

### 2.1. LSTM Feature Extraction Module

Traditional deep learning models often lack engineering-level interpretability. Considering the coupled surrounding-rock-support interaction mechanism on a fully mechanized longwall face, this study introduces an engineering-oriented qualitative interpretation of the LSTM gating behavior. This interpretation is not intended to establish a strict one-to-one physical equivalence between internal gate variables and actual stress states. Instead, it provides an auxiliary perspective for understanding how the LSTM may process pressure accumulation, pressure release, and pressure transfer information in hydraulic-support monitoring sequences.

From this qualitative perspective, the input gate can be regarded as related to the input of newly generated pressure information. In hydraulic-support pressure sequences, this information may correspond to an increase in support working resistance caused by coal cutting, roof rotation, or roof subsidence. Thus, the input gate is interpreted as an auxiliary representation of the pressure-accumulation stage rather than as a direct measurement of stress accumulation. Similarly, the forget gate may be understood as a mechanism for weakening pressure-state information that becomes less relevant to future prediction. In engineering terms, such information may be associated with pressure release caused by support advance, leg lowering, or roof fracture and collapse. However, this relationship should be understood as a model-interpretation analogy, because the forget gate operates on hidden-state information rather than directly measured mechanical stress. The output gate controls the portion of the internal state passed to the hidden representation for subsequent prediction. In support-pressure evolution, this behavior can be qualitatively associated with the transfer and expression of mining-induced pressure information between the surrounding rock and the hydraulic support. This interpretation links sequence learning with the engineering characteristics of roof-support interaction, but does not imply that the output gate directly quantifies stress transfer.

**Figure 1:**
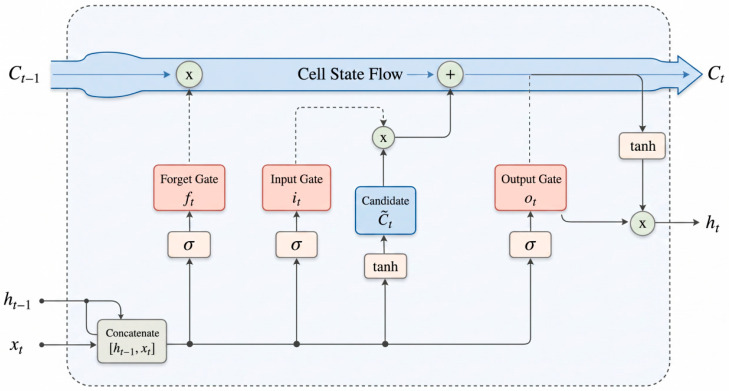
Schematic diagram of the LSTM cell state.

Therefore, the proposed gate interpretation should be regarded as a physically motivated explanatory framework for model behavior rather than as a quantitative mechanical model of stress evolution. Based on this engineering interpretation, intermediate turning features in strata pressure evolution contain important information for future pressure prediction. To avoid losing intermediate states when a standard LSTM outputs only terminal features, the output mode is modified to generate a complete hidden-state sequence with the same length as the historical input window. Its core cell-state update mechanism is expressed as follows. In the experiments, the number of hidden units was set to 7 based on preliminary sensitivity tests and the characteristics of the single-variable support-pressure sequence. Because the input pressure series has a relatively low feature dimension and evolves at a low sampling frequency, an excessive number of recurrent hidden units may introduce parameter redundancy and increase the risk of overfitting. The selected value provided sufficient nonlinear feature representation while maintaining stable training performance and noise suppression. The LSTM cell-state structure is shown in Figure 1.

Cell state update:

(1) $$C_{t}=C_{t-1}⊙f_{t}+g_{t}⊙i_{t}$$

where ⊙ denotes element-wise multiplication.

### 2.2. Transformer Encoding Module

In addition to local dynamic stress evolution, strata pressure time-series data contain periodic roof-weighting patterns induced by continuous face advance. These patterns may recur in similar but nonidentical forms within the historical monitoring sequence. A Transformer encoding module is therefore introduced to characterize such temporal dependencies for future pressure prediction.

**Figure 2:**
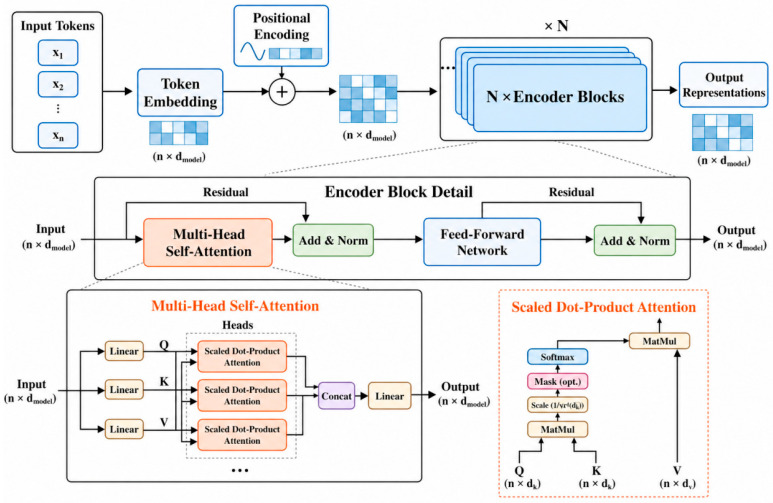
Schematic diagram of the Transformer architecture.

To overcome the limited adaptability of traditional fixed sinusoidal positional encoding to the dynamic evolution of strata pressure, learnable positional embeddings are adopted to learn the relative phase importance of strata pressure across mining cycles. Through the designed two-layer multi-head self-attention mechanism, the model traverses the time dimension and calculates the dynamic weights of different historical pressure states on the current hydraulic-support pressure. This global information aggregation mechanism alleviates information decay in the LSTM and improves the representation of periodic behavior in strata pressure sequences. The Transformer architecture used for global dependency modeling is shown in Figure 2.

### 2.3. Overall Model Architecture

**Figure 3:**
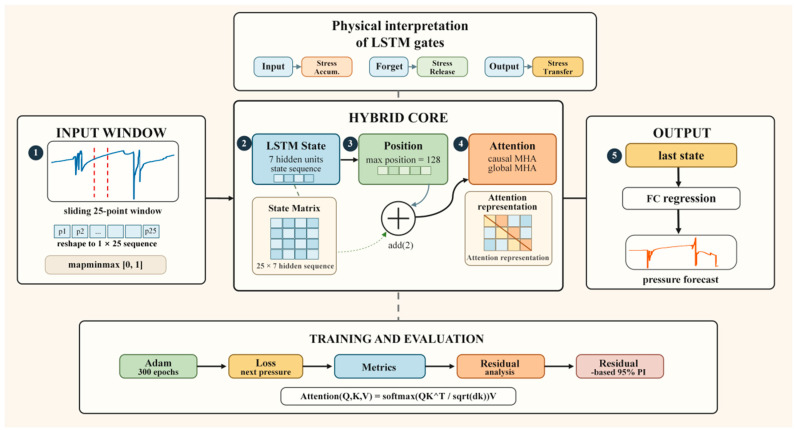
Overall architecture of the proposed model.

To account for both local fluctuations and global dependencies in hydraulic-support pressure dynamics, an LSTM–Transformer hybrid time-series forecasting architecture is constructed, as shown in Figure 3. The model adopts a local–global hierarchical feature extraction strategy, as follows: the LSTM captures local temporal dynamics in the hydraulic-support pressure signal, whereas the Transformer explores global dependencies across time steps. A residual connection is introduced to fuse LSTM-derived local features with positional encoding information, and sequence-end pooling is used to select the terminal global feature for mapping to the final predicted pressure value. The procedure is as follows.

Step 1: For the input hydraulic-support pressure sequence, with the historical window length set to L = 15 in this study, an improved LSTM module first extracts local temporal features and produces the hidden-state sequence, where d = 7 denotes the number of hidden units. The historical input length was selected according to the temporal evolution characteristics of the monitored pressure sequence and preliminary comparisons of different window sizes. A shorter window may not contain sufficient information to describe recent pressure accumulation and release, whereas an overly long window may introduce redundant fluctuations and increase computational burden. Therefore, L = 15 was adopted to balance recent pressure-history representation, prediction accuracy, and model complexity. This process is implemented by Equations (1)–(7) and can be summarized as follows:

(2) $$H_{\mathrm{LSTM}}={\mathrm{LSTM}}_{\mathrm{improved}}(X)$$

Step 2: The hidden-state sequence is then combined with positional embeddings and a residual branch. The positional embedding layer generates a learnable position matrix, with the maximum position index set to 128, which is added elementwise to the hidden-state sequence to obtain Z. The residual branch directly preserves the original local features, which are added to the output of the subsequent block to alleviate gradient vanishing. This process is expressed as follows:

(3) $$Z=H_{\mathrm{LSTM}}+P$$

Step 3: Z is fed into the Transformer encoder. In the first layer, multi-head self-attention is equipped with a causal mask M to ensure that information flows only from historical time steps to future ones. In the second layer, the mask is removed to achieve global information aggregation. Let the number of attention heads be h = 4 and the key dimension be dk = 16. These parameters were determined by considering global dependency modeling ability and computational complexity. Too few attention heads may limit the ability to capture different pressure-evolution patterns, whereas too many may introduce parameter redundancy for the present support-pressure dataset. In preliminary tuning, four attention heads with a key dimension of 16 provided stable prediction performance without substantially increasing the model size. Therefore, h = 4 and dk = 16 were used in the final model. The multi-head attention is computed as follows:

(4) $$\mathrm{MultiHead}(Q,K,V)=\mathrm{Concat}({\mathrm{head}}_{1},\ldots ,{\mathrm{head}}_{h})W^{O}$$

(5) $${\mathrm{head}}_{i}=\mathrm{Attention}(QW_{i}^{Q},KW_{i}^{K},VW_{i}^{V})$$

(6) $$\mathrm{Attention}(Q,K,V)=\mathrm{softmax}\left(\frac{QK^{⊤}}{\sqrt{d_{k}}}+M\right)V$$

The first layer uses masked attention, whereas the second layer is unmasked (M = 0). The outputs of the two layers are denoted by Z1 and Z2, respectively.

Step 4: The hidden state at the last time step of the second-layer output is taken as the representation of the entire sequence. It is then projected through a fully connected linear layer to the output dimension, yielding the future hydraulic-support pressure prediction.

(7) $$\hat{y}=z_{\mathrm{last}}W^{\mathrm{out}}+b^{\mathrm{out}}$$

The model is optimized using the mean squared error (MSE) as the loss function, as follows:

(8) $$L=\frac{1}{N}\sum_{i=1}^{N}{\left(y_{i}-{\hat{y}}_{i}\right)}^{2}$$

where N denotes the number of training samples. Through this procedure, the model establishes a progressive hydraulic-support pressure time-series forecasting framework consisting of local feature extraction, global dependency modeling, and target-value prediction.

## 3. Processing of Measured Pressure Data

### 3.1. Dataset

**Figure 4:**
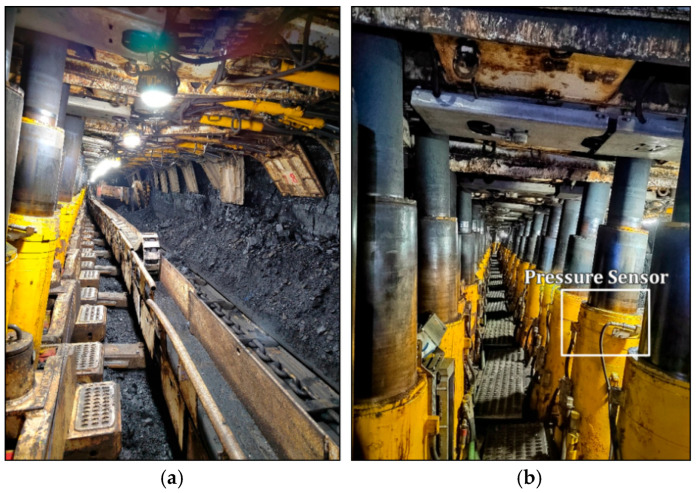
Field monitoring environment and pressure-sensing installation on the hydraulic support. (**a**) Underground layout of hydraulic supports in the 1503 fully mechanized longwall face. (**b**) Local hydraulic-oil pipe and connector region where the pressure sensor is installed.

This study used support-pressure monitoring data from the 1503 fully mechanized mining face of Yili No. 1 Mine over nearly one month, from 12:40 on 1 October 2024, to 23:59 on 28 October 2024. The underground hydraulic-support arrangement and local pressure-sensing installation are shown in Figure 4. In the monitoring system, the pressure sensor was installed at the connector of the hydraulic-oil pipe on the support column circuit, and the collected pressure signal was used to represent the working-resistance response of the hydraulic support. The dataset contains time-synchronized pressure records from representative hydraulic supports, including Support Nos. 10, 40, 70, 149, and 222, and a sample of the raw data structure is presented in Table 1. The sampling interval was 3–5 min. In the modeling stage, the pressure sequence of each support was processed separately, and samples from different support numbers were not concatenated. Support No. 222 was used as a representative case for detailed visualization of prediction results. Considering the sampling interval, the model was configured with a 15-step historical input window to describe recent pressure evolution before the target prediction step. The task of this study is to estimate future mine pressure values from previous support-pressure monitoring records.

### 3.2. Data Cleaning and Preprocessing

In the complex underground production environment, online monitoring data are often contaminated by multiple noise sources, including mining disturbances, electromagnetic interference from equipment, and harsh environmental conditions. If such data are used directly without preprocessing, zero-value patches caused by sensor failure may seriously interfere with model learning of pressure variation patterns, thereby reducing prediction accuracy and stability. Therefore, handling missing values and outliers is a critical prerequisite for pressure forecasting. In this study, missing records were identified when the monitoring system failed to return a valid pressure value at a scheduled sampling instant. Continuous zero-value segments were further examined because support pressure normally remains positive during operation. Zero values occurring only at isolated time points were treated as sensor-recording abnormalities, whereas extended zero-value segments accompanied by unchanged neighboring measurements were considered invalid monitoring records caused by communication interruption or sensor malfunction.

To address missing data, linear interpolation was adopted to impute missing support pressure values [9]. Specifically, known observations before and after each missing point were used to construct an interpolation function, and missing values were estimated according to their relative temporal positions between neighboring observations. Linear interpolation is simple and computationally efficient, and is suitable for support pressure monitoring data with relatively small fluctuations over short time intervals. The interpolation procedure was applied only to short-duration missing intervals. Long continuous missing segments were not reconstructed because they may not reliably reflect the actual pressure-evolution process. This strategy was adopted to preserve the physical characteristics of the original monitoring data as far as possible while improving data integrity and continuity and minimizing distortion of the original trend.

To identify abnormal records, the three-sigma criterion (3σ rule) [10] was used as a supplementary statistical check rather than as a direct deletion criterion. Because high-pressure values in hydraulic-support monitoring may correspond to real periodic roof-weighting events, records exceeding the statistical threshold were compared with the neighboring pressure-evolution trend before processing. Peak values were retained when they showed a continuous rise-and-fall process and remained consistent with the surrounding sequence. Only isolated spikes, discontinuous jumps inconsistent with adjacent records, continuous zero-value segments, and clear sensor-fault records were marked as abnormal observations. The formulas are as follows:

(9) $$\sigma =\sqrt{\frac{\sum_{i=1}^{n}{\left(x_{i}-\bar{x}\right)}^{2}}{n-1}}$$

(10) $$|x_{i}-\bar{x}|>3\sigma$$

According to the statistical results, missing values accounted for approximately 2% of the raw monitoring records, while outliers accounted for about 0.5%. After preprocessing, the number of valid time records in the representative support sequence was 8436. These cleaned records provided the basis for subsequent sliding-window construction of supervised forecasting samples and improved the reliability of model training and testing.

(1) Data normalization

Considering that support-pressure monitoring involves variables with different numerical scales and value ranges, direct model training may adversely affect convergence. Therefore, min–max normalization was applied to map the sample data into the interval [0, 1]. To reduce the risk of future information leakage, the normalization parameters were determined from the training subset and then applied to the test subset. The formula is as follows:

(11) $$x_{\mathrm{norm}}=\frac{x-x_{\min}}{x_{\max}-x_{\min}}$$

where x denotes the raw data, the minimum and maximum values are calculated from the training subset, and the normalized value is denoted by x_norm_.

(2) Time-series partitioning of the dataset

To avoid future data leakage, the dataset was partitioned in chronological order so that the test period contained only later records unseen during model training. For each support-specific experiment, sliding-window sample construction was performed according to the historical input length and target prediction step. Approximately 1185 supervised samples were generated for each support sequence, among which the earliest 948 samples were used for training and the latest 237 samples were used for testing. The value of 1185 therefore refers to the number of supervised samples for a single support sequence after window construction, rather than the number of raw monitoring records or the total number of samples from all supports. This procedure is consistent with the practical scenario of predicting future pressure based on historical records in mine-pressure forecasting [11].

In the experimental process, each hydraulic support was modeled independently using its own pressure sequence. Therefore, the train-test split, normalization parameters, and error metrics were calculated separately for each support. This design avoids artificial information leakage caused by mixing different support positions, while cross-support transfer capability remains a topic for future work.

### 3.3. Model Evaluation Metrics

To comprehensively evaluate model prediction performance, four metrics were selected, as follows: mean absolute error (MAE), mean squared error (MSE), symmetric mean absolute percentage error (SMAPE), and coefficient of determination (R^2^). These metrics assess prediction accuracy and reliability from different perspectives.

(1) Mean absolute error $E_{\mathrm{MAE}}$

$E_{\mathrm{MAE}}$ is the average of the absolute differences between the true values and the predicted values, and is defined as follows:

(12) $$E_{\mathrm{MAE}}=\frac{1}{n}\sum_{i=1}^{n}\left|x-\hat{x}\right|\;$$

where x denotes the true value, $\hat{x}$ denotes the predicted value, and n denotes the sample size. $E_{\mathrm{MAE}}$ reflects the average level of prediction error. A smaller value indicates higher prediction accuracy of the model.

(2) Mean squared error $E_{\mathrm{MSE}}$

$E_{\mathrm{MSE}}$ is the average of the squared differences between the true values and the predicted values, and is defined as follows:

(13) $$E_{\mathrm{MSE}}=\frac{1}{n}\sum_{i=1}^{n}{\left(x-\hat{x}\right)}^{2}\;$$

Because $E_{\mathrm{MSE}}$ assigns greater weight to larger errors, it more effectively reflects large deviations in model prediction.

(3) Symmetric mean absolute percentage error $E_{\mathrm{sMAPE}}$

$E_{\mathrm{sMAPE}}$ is a metric commonly used to evaluate the performance of time-series forecasting models. Its main advantage lies in measuring the relative error between predicted values and true values. The formula is as follows:

(14) $$E_{\mathrm{sMAPE}}=\frac{100\%}{n}\sum_{i=1}^{n}\frac{\left|x_{i}-\hat{x_{i}}\right|}{\frac{\left|x_{i}\right|+\left|\hat{x_{i}}\right|}{2}}\;$$

A lower $E_{\mathrm{sMAPE}}$ indicates higher prediction accuracy. Another advantage of $E_{\mathrm{sMAPE}}$ is that it is a dimensionless metric, which allows comparison across datasets of different scales.

(4) Coefficient of determination $R^{2}$

$R^{2}$ is the coefficient of determination, which measures the relationship between the variance explained by the model predictions and the total variance of the true values. It is calculated as follows:

(15) $$R^{2}=1-\frac{\sum_{i=1}^{n}{\left(x_{i}-\hat{x_{i}}\right)}^{2}}{\sum_{i=1}^{n}{\left(x_{i}-\bar{x}\right)}^{2}}\;$$

where the mean of the true values is denoted by x-bar. R^2^ ranges from 0 to 1, and a value closer to 1 indicates stronger predictive capability, meaning that the model can better capture variation patterns in the data.

MAE and MSE directly reflect the average prediction error of the model, SMAPE describes the relative error magnitude, and R^2^ reflects the ability of the model to explain data variability and thus evaluates goodness of fit. Although these metrics provide important information about model performance, each has its limitations, as follows: MAE and MSE may be sensitive to outliers, whereas SMAPE may become unstable when values are close to 0. Therefore, a comprehensive evaluation based on these four metrics was adopted to assess the overall performance of the model.

## 4. Investigation of Support Pressure Prediction with the LSTM–Transformer Model

### 4.1. Effect of Forecast Horizon on Prediction Accuracy

To evaluate model performance under different forecast horizons, four forecasting horizons were considered in the experiments, namely 15, 20, 25, and 30 time steps. For each horizon, the model predicts the target pressure value at the corresponding future offset, and the rolling prediction curves were obtained by moving the historical input window forward in chronological order. These settings cover forecasting needs from short to relatively long horizons, enabling assessment of the model’s applicability and robustness across different prediction tasks. The performance of the evaluation metrics is listed in Table 2.

**Figure 5:**
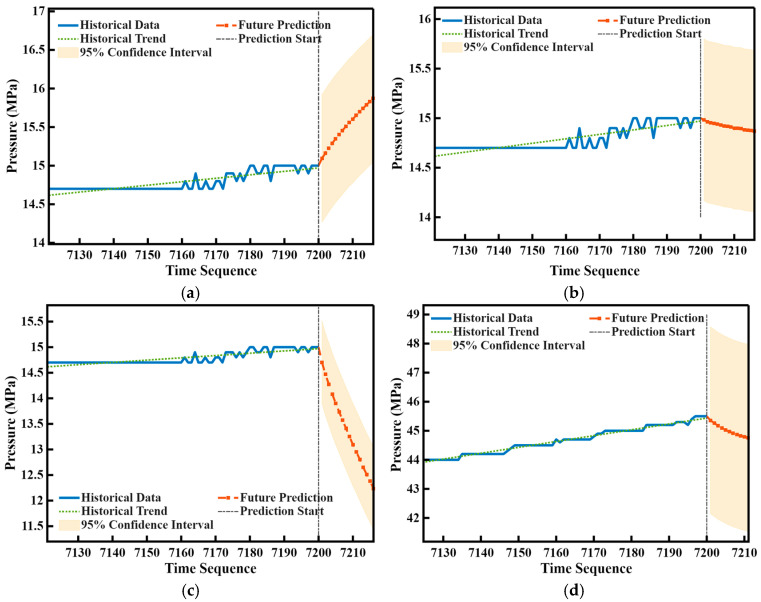
Time-series forecasting results with residual-based prediction intervals under different forecast horizons. (**a**) Step size = 15; (**b**) Step size = 20; (**c**) Step size = 25; (**d**) Step size = 30.

The residual-based prediction interval in Figure 5 was constructed from the residual distribution of the training set. Specifically, the residual standard deviation was calculated after model training, and the prediction interval was expressed as the point prediction plus or minus 1.96 times the residual standard deviation. This interval serves as an auxiliary indication of prediction dispersion rather than as a probabilistic early-warning threshold.

### 4.2. Effect of Data Volume on Prediction Accuracy

**Figure 6:**
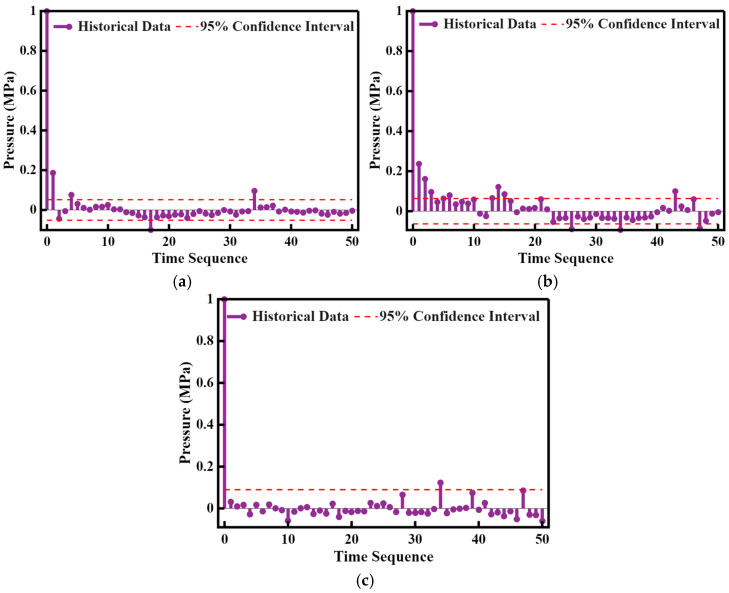
Error autocorrelation analysis. (**a**) 10 days; (**b**) 20 days; (**c**) 30 days.

**Figure 7:**
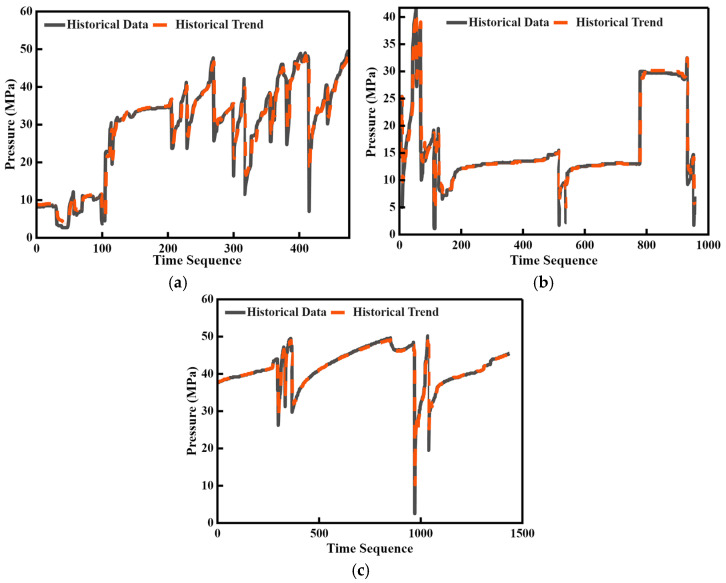
Comparison of support pressure prediction under different data volumes. (**a**) 10 days; (**b**) 20 days; (**c**) 30 days.

To investigate the effect of training data volume on prediction accuracy, error autocorrelation analyses were performed using datasets covering 10 days, 20 days, and the full-period dataset of approximately 30 days, respectively. Figure 6 shows the autocorrelation function (ACF) of the forecast errors, with the dashed lines indicating the 95% confidence interval. The results show that when the training data volume is only 10 days, the error series exhibits significant autocorrelation at multiple lag orders, with ACF values exceeding the confidence interval, indicating that the model has not fully captured the dynamic pattern of the data and that systematic prediction components remain in the residuals. When the training data volume increases to 20 days, the error autocorrelation is weakened; at most lag orders, the residuals fluctuate within the confidence interval, indicating that the model fit has improved. When the data volume further increases to the full-period dataset, the error autocorrelation becomes weaker overall and approaches a white-noise distribution, suggesting that the model has more adequately learned the intrinsic structure of the sequence and that the prediction residuals are closer to random disturbances. This demonstrates that an increase in training data volume helps improve the stability and accuracy of the model and enhances its ability to characterize the evolution of support pressure. The corresponding prediction curves under different data volumes are shown in Figure 7.

The results show that increasing the data volume did not lead to monotonic improvement in performance, but instead showed a clear threshold effect. When the data volume increased from 10 to 20 days, the improvement in model performance was limited, as follows: EMSE decreased only slightly from 2.83 to 2.81, and R^2^ increased marginally from 0.943 to 0.945, whereas EMAE increased from 0.92 to 1.07 and ESMAPE rose from 3.51% to 3.72%. This indicates that, at a moderate data scale, the model remained constrained by sample noise or local fluctuations and could not stably capture complete dynamic patterns in the time series. However, when the data volume reached the full-period dataset, model performance improved substantially, as follows: EMSE decreased markedly to 2.42, EMAE improved to 0.70, and R^2^ increased to 0.972, with the goodness of fit approaching the ideal level. Although ESMAPE increased slightly to 3.76%, the overall error remained low, and the comprehensive improvement in prediction accuracy indicates enhanced model generalization within the tested support sequence.

This nonlinear growth pattern reveals an intrinsic relationship between data scale and the modeling of periodic mine-pressure behavior. Monitoring data spanning 10 or 20 days may cover only one to two mining-induced pressure cycles in the temporal dimension, making the model prone to local overfitting and to mistaking random fluctuations under specific geological conditions for global patterns. In contrast, the full-period dataset provides more complete temporal coverage of the initial weighting stage and multiple periodic weighting cycles of the working face. At this point, the data scale exceeds the threshold required to capture a complete roof-movement cycle, enabling the self-attention mechanism of the Transformer module to more fully compare stress-evolution precursors across multiple pressure cycles and thereby improve generalization performance. The non-monotonic changes in evaluation metrics with increasing data volume can also be explained by pressure-cycle coverage and sample heterogeneity. When only 10 days of data were used, the training set contained limited pressure-evolution patterns, so the model tended to fit local fluctuations within a short mining stage. Increasing the data volume to 20 days introduced more samples, but these additional samples did not necessarily provide complete and consistent roof-weighting cycles. Instead, they may have included transitional mining stages, local disturbances, or pressure-release processes that differed from the earlier samples. As a result, the error metrics did not improve monotonically. When the full-period dataset was used, the data covered more complete pressure accumulation, peak development, and pressure-release processes, allowing the model to learn more stable temporal dependencies and reduce systematic residual autocorrelation. Larger errors were mainly observed near abrupt pressure-rise and pressure-drop segments. During these periods, pressure changes rapidly within a few sampling intervals, whereas the model output tends to be smoother because it is learned from historical-window patterns. This may cause local phase lag near the beginning of a pressure peak and underestimation or overestimation near rapid pressure-release stages. Therefore, the prediction results are more reliable for describing short-term pressure evolution trends under relatively continuous pressure variation, whereas individual predictions near sudden roof-weighting or operation-induced pressure disturbances should be interpreted with caution.

### 4.3. Effect of Initial Learning Rate on Prediction Accuracy

Training parameters play a critical role in model convergence and prediction accuracy. Before the final model evaluation, preliminary hyperparameter tuning experiments were conducted to determine appropriate architectural and training parameters. The candidate parameters included the historical input window length, the number of LSTM hidden units, the number of attention heads, the key dimension of the attention module, the initial learning rate, the batch size, and the maximum number of training epochs. The final parameter combination was selected by jointly considering prediction accuracy on the validation subset, convergence stability, and model complexity. Validation MAE and MSE were used as the primary indicators, while overly complex parameter settings were not adopted when they did not produce clear improvements in prediction performance. In this study, the Adam optimizer was adopted throughout the learning-rate sensitivity experiment. Adam combines the advantages of Adagrad and RMSprop, enables adaptive adjustment of the learning rate, and accelerates convergence, and is therefore suitable for complex network architectures. To explore the performance of the LSTM–Transformer hybrid model on support pressure data, the maximum number of training epochs was set to 300, and a staged learning-rate decay strategy was applied, with the learning rate reduced to 0.1 times its original value at the 150th epoch. The final model used a historical input length of 15, seven LSTM hidden units, four attention heads, a key dimension of 16, an initial learning rate of 0.005, a batch size of 32, and a maximum of 100 training epochs. This setting achieved a good balance between prediction accuracy and convergence stability in the preliminary tuning process. Further increasing the number of recurrent hidden units or attention heads did not clearly improve validation performance but increased model complexity. Similarly, the selected batch size and epoch number allowed stable convergence while avoiding unnecessary repeated training.

**Figure 8:**
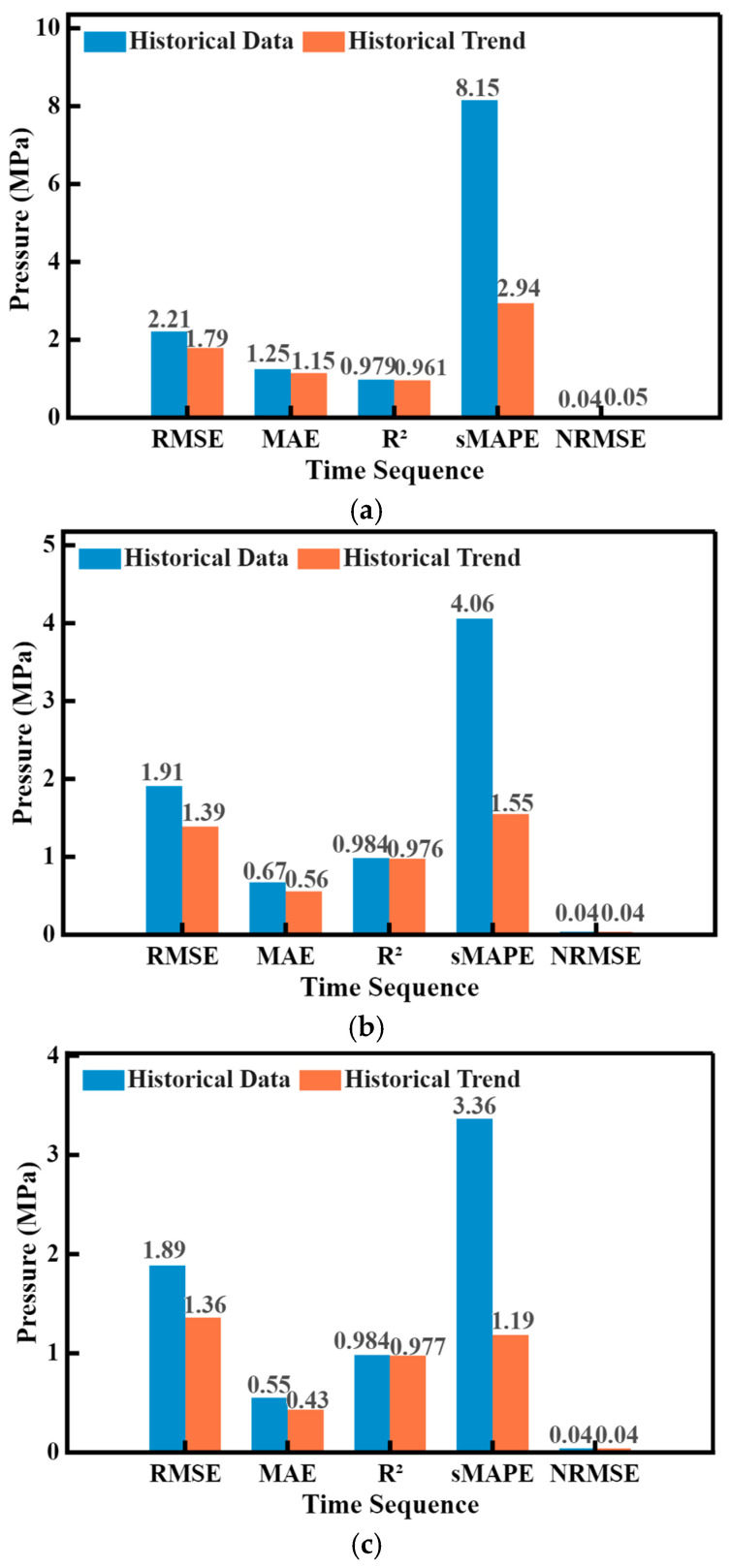
Performance metrics under different learning rates. (**a**) Learning rate = 0.1; (**b**) Learning rate = 0.01; (**c**) Learning rate = 0.001.

A specific architecture configuration was adopted for the model, as follows: the number of hidden units in the LSTM layer was 7; the Transformer component included 4 attention heads, with a key-channel dimension of 64 and a maximum positional encoding length of 128; and the dropout rate was set to 0.01. Considering computational resource constraints in deep learning training, the batch size was fixed at 16 to ensure both training stability and storage efficiency. To evaluate model performance under different initial learning rates, three learning rates were tested, namely 0.1, 0.01, and 0.001, while the optimizer was kept unchanged. The results are summarized in Figure 8.

The results show that the initial learning rate has a significant effect on model performance. When the learning rate was 0.1, the model was able to converge, but its test-set performance was relatively poor, with RMSE = 1.79, MAE = 1.15, R^2^ = 0.961, and SMAPE = 2.94%. This indicates that an excessively large step size may cause oscillation near the optimum and prevent precise convergence. When the learning rate decreased to 0.01, model performance improved significantly, as follows: test RMSE decreased to 1.39, MAE decreased to 0.56, R^2^ increased to 0.976, and SMAPE decreased to 1.55%. This suggests that an appropriate learning rate can effectively reduce error while ensuring stable convergence toward the optimal solution.

When the learning rate was further reduced to 0.001, the model achieved the best test-set performance among the three settings, as follows: RMSE decreased to 1.36, MAE was further reduced to 0.43, R^2^ remained high at 0.977, and SMAPE decreased to 1.19%. These results indicate that a finer step size enables the model to explore local details of the loss surface more fully and achieve more accurate fitting.

These trends reveal the critical balancing role of the learning rate in time-series model training. An excessively high learning rate leads to coarse convergence, whereas a lower learning rate allows the optimization trajectory to enter the optimal region more smoothly. Accordingly, a learning rate of 0.001 was preferred in the subsequent experiments to maximize prediction accuracy while ensuring stable error control.

### 4.4. Model Comparison

To comprehensively evaluate the effectiveness of the proposed prediction model in practical support pressure forecasting, comparative experiments were conducted against three representative baseline models, as follows: the classical time-series forecasting model LSTM; the convolutional neural network-long short-term memory model (CNN-LSTM), which incorporates convolutional structures to extract local features; and the random forest model, an ensemble learning approach widely used in mining engineering. All models used the same chronological train-test partition, normalization strategy, and support-specific experimental protocol. By comparing the predictive performance of these models under the same experimental conditions, the advantages of the proposed model in prediction accuracy, test-set generalization, and robustness were evaluated. The comparison results are as follows.

**Figure 9:**
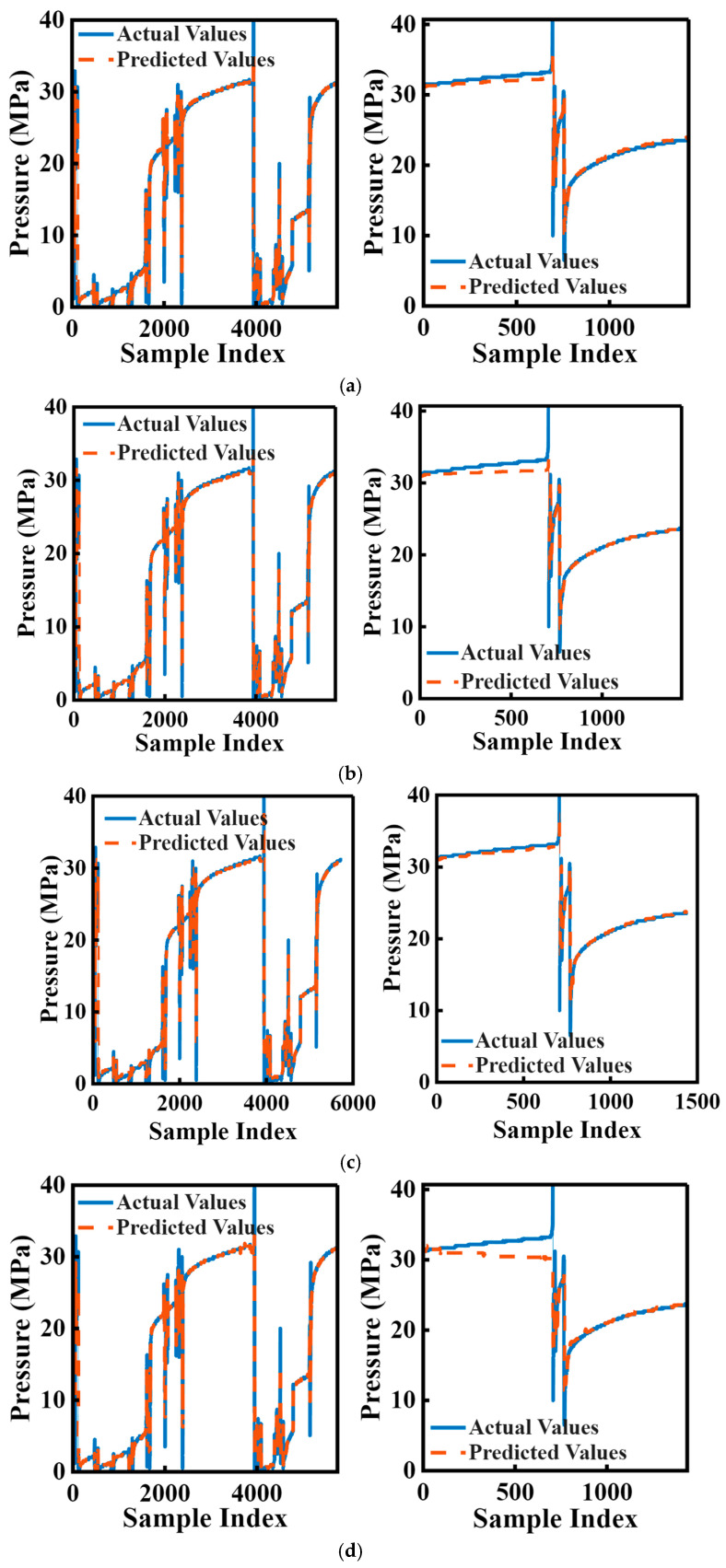
Comparison of support pressure prediction under different models. (**a**) LSTM; (**b**) CNN-LSTM; (**c**) LSTM–Transformer; (**d**) Random Forest.

Figure 9 presents the prediction results of the four models on the training and test sets. The left subplots show fitting performance on the training set, whereas the right subplots show generalization performance on the test set. The solid line represents the actual support pressure values, and the dashed line represents model predictions.

On the training set, all four models captured the overall trend of the pressure sequence, but with clear differences in accuracy. The conventional LSTM extracted temporal dynamics but exhibited phase lag under sharp pressure fluctuations, indicating limited capability in local detail characterization. CNN-LSTM improved fitting performance by extracting local features through convolution, but deviations still appeared in peak regions. Random forest reproduced the overall trend but produced a relatively smooth prediction curve and lacked sufficient ability to reproduce fine-scale fluctuations. In contrast, the proposed LSTM–Transformer model achieved the best performance, with its predicted curve nearly overlapping the actual values, especially in regions of abrupt pressure variation. This superiority arises from effective global-information modeling by the self-attention mechanism and its strong complementarity with the temporal modeling capability of LSTM.

On the test set, differences in prediction performance among the models became more pronounced. The prediction error of LSTM increased significantly, with obvious lag during periods of intense fluctuation. CNN-LSTM showed some improvement but still struggled to reproduce detailed oscillations. Random forest remained relatively stable, yet its predictions were overly smooth and failed to recover extreme values accurately. The LSTM–Transformer model maintained the best prediction performance in the tested support sequence, showing high agreement with the actual pressure curve and smaller errors in trends, peaks, and turning points, thereby confirming its stronger capability in handling the nonlinear and nonstationary characteristics of support pressure sequences.

**Figure 10:**
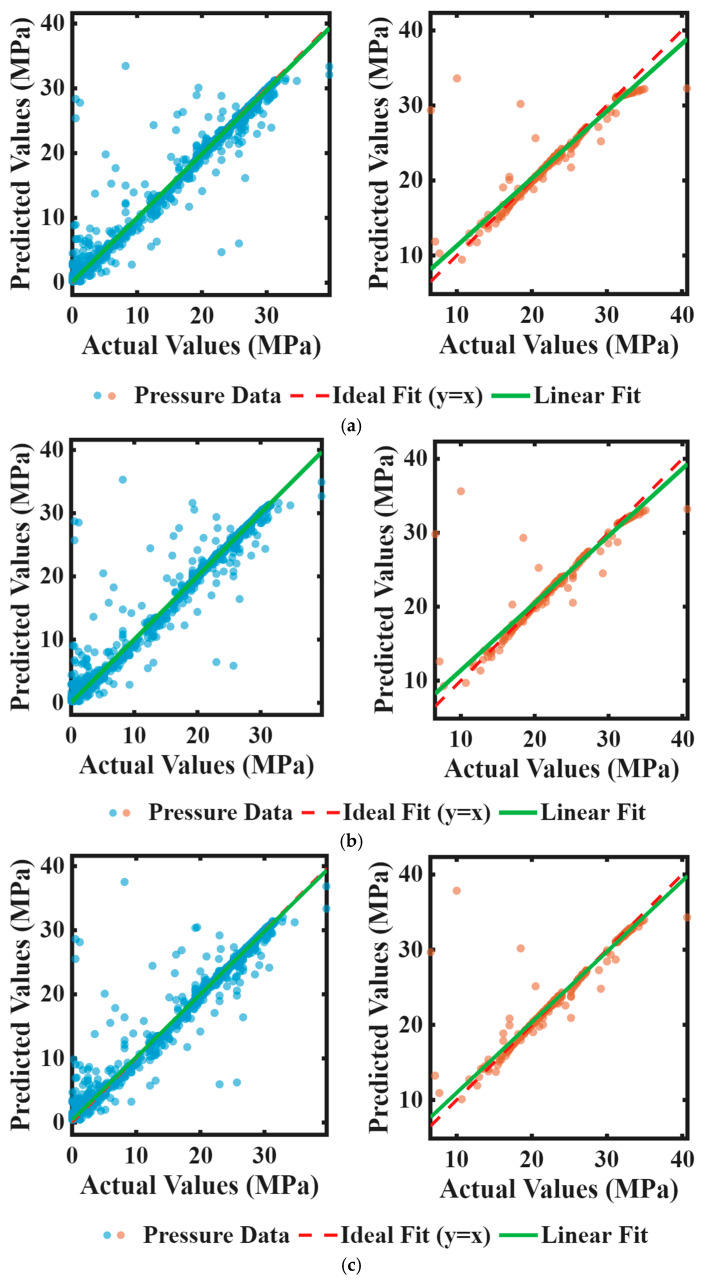

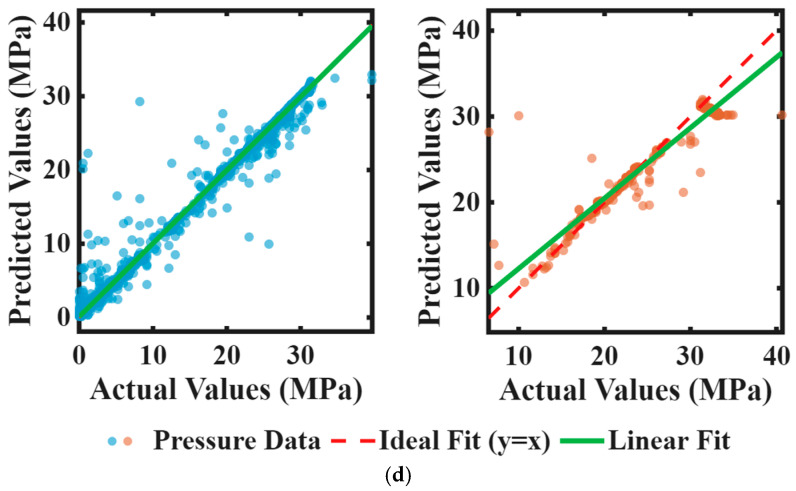
Scatter-point comparison of support pressure prediction under different models. (**a**) LSTM; (**b**) CNN-LSTM; (**c**) LSTM-Transformer; (**d**) Random Forest.

Figure 10 shows the scatter-plot comparison between predicted and actual values for different models on the training and test sets.

Figure 10 compares the prediction performance of LSTM, CNN-LSTM, LSTM–Transformer, and random forest on the training and test sets. The proposed LSTM–Transformer model achieved the best fitting accuracy and test-set performance during both training and testing. Its scatter points are densely distributed near the ideal line (y = x), and the fitted regression line almost coincides with the ideal line. Compared with baseline LSTM, the proposed model effectively alleviates information attenuation in long-range dependencies, resulting in smaller prediction deviations on the test set. Compared with CNN-LSTM, the self-attention mechanism enables more accurate capture of global dependencies and further reduces error dispersion. Compared with random forest, the proposed model benefits from hierarchical feature learning in deep neural networks and maintains a highly concentrated point distribution on the test set, demonstrating stronger performance than conventional ensemble learning methods under the same experimental conditions. These comparisons indicate that the LSTM–Transformer model has clear advantages in both prediction accuracy and stability for the tested support-pressure sequence.

The comprehensive comparison results demonstrate that the proposed LSTM–Transformer hybrid prediction model outperforms conventional LSTM, CNN-LSTM, and random forest models in both fitting accuracy and test-set prediction capability for support pressure sequences. Relative to standalone LSTM, the Transformer self-attention mechanism compensates for insufficient long-range dependency modeling and reduces phase lag and peak deviation in regions with intense pressure fluctuations. Compared with CNN-LSTM, the proposed model overcomes the limitation of local receptive fields and adaptively fuses global temporal associations, thereby improving the reconstruction of detailed fluctuations and key turning points. Compared with the piecewise fitting and smoothing characteristics of random forest, the LSTM–Transformer model provides stronger nonlinear mapping capability and temporal feature-mining ability, enabling more accurate capture of abrupt changes and extreme characteristics in support pressure. The superior performance of the model arises from the complementarity between efficient local temporal modeling by LSTM and global contextual feature extraction by the Transformer. As a result, the model demonstrates stronger predictive reliability when dealing with nonlinear and nonstationary field-measured support pressure data.

### 4.5. Ablation Study

To further examine the contribution of the main components in the proposed architecture, an ablation study was conducted using the same representative support sequence, chronological train-test split, normalization strategy, training settings, and evaluation metrics. The full LSTM–Transformer model was compared with several simplified variants, including the LSTM-only model, Transformer-only model, and the LSTM–Transformer model without learnable positional encoding. In addition, the effect of the residual connection was examined during preliminary ablation testing.

The ablation results show that the full LSTM–Transformer model achieved the best overall prediction performance among the stable tested variants, especially in terms of MAE and SMAPE. Compared with the LSTM-only model, the full model reduced MAE from 0.3234 MPa to 0.3034 MPa and SMAPE from 1.22% to 1.00%, indicating that the Transformer module improves local prediction accuracy and relative-error control after LSTM-based temporal feature extraction. Compared with the Transformer-only model, the full model achieved lower RMSE, MAE, and SMAPE, suggesting that the LSTM module is necessary for capturing local nonlinear pressure-evolution characteristics before global dependency modeling.

The model without learnable positional encoding obtained a slightly lower RMSE than the full model, but its MAE and SMAPE were higher. This indicates that removing positional encoding may reduce some point-wise squared errors in this test case but weakens the ability of the model to control average and relative prediction errors. Therefore, learnable positional encoding was retained in the final architecture to provide explicit temporal-position information for the attention mechanism.

In addition, preliminary ablation testing showed that removing the residual connection led to unstable convergence and obvious deterioration in prediction results. Therefore, this variant was not listed as a stable comparable model in Table 3. This result indicates that the residual connection plays an important role in preserving LSTM-derived local temporal features and stabilizing Transformer-based feature transformation.

## 5. Conclusions

To address the nonlinear and nonstationary characteristics of mine pressure evolution in a fully mechanized mining face, an LSTM–Transformer hybrid time-series forecasting model integrating local dynamic perception and global dependency modeling was proposed and validated using field-measured hydraulic-support pressure data. The main conclusions are as follows.

(1) The LSTM gating mechanism provides an engineering-oriented interpretation related to stress accumulation, release, and transfer, whereas the Transformer self-attention mechanism addresses the limitation of conventional temporal modeling in jointly characterizing local pressure mutations and global periodic weighting. The experimental results show that, under the optimal parameter configuration of a learning rate of 0.001 and a full-period training dataset, the hybrid model achieved strong test-set performance with R^2^ = 0.971 and reduced the phase-lag effect in pressure peak regions.
(2) Sufficient historical data are a prerequisite for effective global modeling of mine pressure sequences. When the training set fully covers periodic weighting cycles and is combined with an appropriate learning rate, the self-attention mechanism can compare historical pressure-evolution patterns across cycles, thereby improving model generalization and reducing systematic residual components.
(3) Under multi-step rolling forecasting, the model maintained low error and high stability within 25 steps, corresponding to approximately 1–2 h under the sampling interval used in this study. When the forecasting horizon was further extended, the nonlinear evolution of roof movement and support response caused more obvious error accumulation. Therefore, the 25-step case provides a useful reference range for future mine pressure prediction in the present dataset.

Although satisfactory results were achieved in pressure time-series forecasting, practical deployment of the proposed model should still consider underground engineering conditions. Mine pressure evolution is jointly affected by geological structure, roof lithology, support posture, neighboring support interaction, face advance rate, and mining-induced dynamic disturbances. Therefore, in real monitoring systems, the proposed model is more suitable as a support-pressure forecasting module that provides short-term reference information for abnormal pressure increase, delayed pressure release, and pressure-peak development, while warning decisions should be made together with engineering thresholds and multi-source monitoring information.

Future work will embed the LSTM–Transformer forecasting model into a multi-source intelligent mine-pressure monitoring framework by incorporating multi-support pressure fields, hydraulic-support attitude information, advancing speed, roof lithology, and microseismic signals. Considering the difficulty of obtaining continuous and high-quality underground field data, model training and testing in this study were conducted using data from one representative working face over a specific monitoring period. Additional datasets from different working faces, mining stages, hydraulic-support types, and operational conditions will be collected to further evaluate cross-support transferability, cross-condition generalization, and engineering applicability.

## Figures and Tables

**Table 1:** Example of raw data.

Time	Pressure/MPa					
	10	40	70	…	149	222
2024–10–1T 12:40	11	22	14.1	…	6.6	29
2024–10–1T 12:43	13.2	24.4	14.5	…	6.6	28.7
2024–10–1T 12:47	14.5	26	15	…	6.7	28.5
2024–10–1T 12:50	15.2	29.5	16	…	6.6	28.3
…	…	…	…	…	…	…
2024–10–28T 12:52	23.7	15	4.2	…	18.6	25.5
2024–10–28T 12:55	23.5	15	4.2	…	18.6	25.5
2024–10–28T 23:59	23.7	15	4.2	…	18.7	25.5

**Table 2:** Evaluation metrics under different forecast horizons.

Step	Evaluation Metrics			
	RMSE/MPa	MAE/MPa	R^2^	SMAPE/%
15	1.100	0.280	0.964	1.160
20	1.090	0.220	0.964	0.960
25	1.090	0.200	0.964	0.900
30	1.200	0.580	0.957	2.190

**Table 3:** Ablation study of the proposed model.

Model Variant	Model Components				Evaluation Metrics			
	LSTM	Trans.	PE	Res.	RMSE /MPa	MAE /MPa	R^2^	SMAPE /%
LSTM only	✓	–	–	–	1.1227	0.3234	0.9621	1.22
Transformer only	–	✓	✓	✓	1.1612	0.4747	0.9594	1.86
LSTM–Transformer without positional encoding	✓	✓	–	✓	1.0975	0.3731	0.9630	1.53
Full LSTM–Transformer	✓	✓	✓	✓	1.1222	0.3034	0.9635	1.00

## Data Availability

The data can be provided upon request by the authors.
